# Freedom From Seizures Might Be Key to Continuing Occupation After Epilepsy Surgery

**DOI:** 10.3389/fneur.2021.585191

**Published:** 2021-02-12

**Authors:** Toshiki Nozaki, Ayataka Fujimoto, Tomohiro Yamazoe, Keiko Niimi, Shimpei Baba, Takamichi Yamamoto, Keishiro Sato, Hideo Enoki, Tohru Okanishi

**Affiliations:** Comprehensive Epilepsy Center, Seirei Hamamatsu General Hospital, Hamamatsu, Japan

**Keywords:** occupation continuity, seizure freedom, epilepsy surgery, intelligence quotient, employment

## Abstract

**Introduction:** We hypothesized that epilepsy surgery for adult patients with temporal lobe epilepsy (TLE) who obtained freedom from seizures could provide opportunities for these patients to continue their occupation, and investigated continuity of occupation to test this postulation.

**Methods:** Data were obtained from patients who had undergone resective surgery for medically intractable TLE between October 2009 and April 2019 in our hospital. Inclusion criteria were as follows: (1) ≥16 years old at surgery; (2) post-operative follow-up ≥12 months; (3) seizure-free period ≥12 months. As a primary outcome, we evaluated employment status before and after surgery, classified into three categories as follows: Level 0, no job; Level 1, students or homemakers (financially supported by a family member); and Level 2, working. Neuropsychological status was also evaluated as a secondary outcome.

**Results:** Fifty-one (87.9%) of the 58 enrolled TLE patients who obtained freedom from seizures after surgery continued working as before or obtained a new job (employment status: Level 2). A significant difference in employment status was identified between before and after surgery (*p* = 0.007; Wilcoxon signed-rank test). Twenty-eight patients (48.3%) were evaluated for neuropsychological status both before and after surgery. Significant differences in Wechsler Adult Intelligence Scale-III scores were identified between before and after surgery (*p* < 0.05 each; paired *t*-test).

**Conclusion:** Seizure freedom could be a factor that facilitates job continuity, although additional data are needed to confirm that possibility. Further investigation of job continuity after epilepsy surgery warrants an international, multicenter study.

## Introduction

A surgical strategy is currently considered preferable for treating drug-resistant temporal lobe epilepsy (TLE) (1, 2), and physicians and patients thus frequently choose surgical treatments for drug-resistant TLE. Some authors have suggested that the unemployment rate is reduced after epilepsy surgery (3–5). We therefore theorized that the surgical strategy should be chosen more frequently for patients with intractable TLE. However, many patients with intractable TLE hesitate to undergo surgery when offered this treatment option. This hesitation is probably because surgery may be associated with cognitive and other permanent changes (6). For example, one report about occupations of epilepsy patients reported that the ability to continue an occupation at the same level was a critical issue for patients such as musicians (7). Consequently, physicians need to consider whether patients will be able to continue their regular activities post-operatively.

A large amount of research into employment and unemployment of individuals with epilepsy already exists (8–13), along with research examining intelligence quotient (IQ) in children with epilepsy (14–20). However, clear answers have been lacking regarding whether patients can continue their occupation post-operatively (12). Job continuity might thus be an important factor that physicians should take into account. In this study, we hypothesized that epilepsy surgery for patients with TLE who obtained freedom from seizures would provide opportunities to continue their occupation. The purpose of this study was to investigate continuity of occupation in adult patients with TLE.

## Methods

### Patients

Data were obtained from all 78 patients who had undergone resective surgery for medically intractable TLE between October 2009 and April 2019 in our hospital. Of these, 58 patients (24 women, 34 men) fulfilled the following inclusion criteria: 1) age ≥16 years at surgery; 2) follow-up ≥12 months after surgery; 3) a seizure-free (International League Against Epilepsy classification 1: completely seizure-free, no auras) (21) period ≥12 months during follow-up after surgery. Twelve patients <15 years old, six patients who could not obtain freedom from seizures, and three patients who did not fulfill the follow-up criterion were excluded from the primary outcome measurement, but were still analyzed for secondary outcomes.

### Epilepsy Surgery

All patients received standardized pre-operative evaluations, comprising electroencephalography (EEG), magnetic resonance imaging (MRI), long-term video-EEG, interictal 2-[^18^F]fluoro-2-deoxy-D-glucose positron emission tomography (^18^FDG-PET), iomazenil-single-photon emission computed tomography (IMZ-SPECT) and the Wada test, and had been diagnosed with intractable TLE. Based on the results of these examinations, neuropsychological assessment and employment status of every patient, appropriate treatment strategies were discussed at conferences in our comprehensive epilepsy center, with the participation of pediatric neurologists, neurologists, neurosurgeons, neurophysiologists, and neuropsychologists. Some patients with MRI-negative findings, possible bilateral temporal lobe onset, or involvement of a large area of neocortex underwent subdural grid electrode placement and depth electrode insertion on the appropriate region, as necessary. All patients underwent one of the following resective surgeries: anterior temporal lobectomy; hippocampal transection; selective amygdalohippocampectomy; or focus resection. All resected specimens were histopathologically examined except for the case of hippocampal transection.

### Primary Outcome Measurement—Employment Status and Occupations

We evaluated employment status in all patients preoperatively and ≥12 months after resective surgery. Employment status was classified into the following 3 categories: Level 0, no job; Level 1, students or homemakers (financially supported by a family member); or Level 2, working. The post-operative employment status used for this study was recorded at the time of last follow-up evaluation at an outpatient clinic.

In accordance with the 2008 ISCO (ISCO-08) by the International Labor Organization, we divided occupations into 10 major groups: 1) Managers; 2) Professionals; 3) Technicians and associate professionals; 4) Clerical support workers; 5) Service and sales workers; 6) Skilled agricultural, forestry and fishery workers; 7) Craft and related trade workers; 8) Plant and machine operators, and assemblers; 9) Elementary occupations; and 10) Armed forces occupations. As housemakers, students, and the unemployed cannot be classified by ISCO-08, we listed these independently.

### Secondary Outcome Measurements

#### Neuropsychological Assessment

Neuropsychological status was evaluated by both the Wechsler Adult Intelligence Scale-III (WAIS-III) and the Wechsler Memory Scale-revised (WMS-R) preoperatively and ≥3 months after resective surgery. From among the 58 patients enrolled in this study, we assessed changes in cognitive function as evaluated by WAIS-III [verbal IQ (VIQ), performance IQ (PIQ), full-scale IQ (FIQ)] and memory function as evaluated by WMS-R before and after surgery in 28 patients (48.3%) who were able to be evaluated both preoperatively and ≥3 months after resective surgery.

#### Comparison Between Patients With Seizure Freedom and With Residual Seizures

Employment status before and after surgery was compared between patients with seizure freedom compared to a reference group with residual seizures.

### Statistical Analysis

Data from employment status were statistically analyzed. Comparison of employment status before and after surgery was also analyzed by Wilcoxon signed-rank test. Age at seizure onset and seizure frequency for each employment status prior to resective surgery were compared using one-way analysis of variance (ANOVA). We used paired *t*-tests for comparisons of scores acquired by WMS-R and WAIS-III before and after surgery as neuropsychological examinations. Values of *p* < 0.05 were considered to indicate significant differences in all analyses. Easy R (EZR) statistical software (Saitama Medical Center, Jichi Medical University, Saitama, Japan) was used for all statistical analyses.

### Ethics Approval

The ethics committee at our hospital approved this study protocol in accordance with the principles of the Declaration of Helsinki (permission number: 3349).

## Results

### Patient Characteristics

We investigated 58 patients who fulfilled our inclusion criteria. Table 1 shows the baseline characteristics of the enrolled patients.

**Table 1:** Baseline characteristics (*N* = 58).

Sex (female)	24 (41.4%)
Seizure duration, years	15.1 ± 12.0
Seizure onset, years	18.8 ± 10.3
Age at surgery, years	33.9 ± 11.0
Follow-up, months	43.7 ± 28.4
**Language dominance**	
Left	52 (89.7%)
Right	6 (10.3%)
**Affected side**	
Left	27 (46.%)
Right	31 (53.4%)
**Epilepsy surgery**	
Anterior temporal lobectomy	38 (65.5%)
Anterior temporal lobectomy + focus resection	9 (15.5%)
Selective amygdalohippocampectomy	5 (8.6%)
Selective amygdalohippocampectomy + focus resection	3 (5.1%)
Focus resection	2 (3.4%)
Hippocampal transection	1 (1.7%)
VNS therapy required	5 (8.6%)
**Histopathology**	
FCD	13 (22.4%)
HS	11 (19%)
Mild MCD	14 (24.1%)
HS + (FCD or MCD)	7 (12.1%)
Tumor	3 (5.2%)
Cerebrovascular disease	3 (5.2%)
Trauma	2 (3.4%)
Polymicrogyria	1 (1.7%)
Non-specific	3 (5.2%)

*Data are presented as n (percentage) or mean ± standard deviation*.

*VNS, vagus nerve stimulation; FCD, focal cortical dysplasia; HS, hippocampal sclerosis; MCD, malformation of cortical development*.

Only one patient experienced surgical complications, as intracranial hemorrhage, the day after left anterior temporal lobectomy, and underwent evacuation of the hematoma. Forty-four patients had intracranial electrodes inserted to determine the extent of surgical resection with no surgical complications. Two patients had undergone vagus nerve stimulation (VNS) therapy before resective surgery, and three patients underwent implantation of a generator for VNS after resective surgery, because post-operative seizure control proved insufficient. All patients with VNS therapy obtained freedom from seizures for ≥12 months. Age at seizure onset and seizure duration showed no significant effects on employment status prior to resective surgery (*p* = 0.417, *p* = 0.655, respectively).

### Occupational Continuity as a Primary Outcome Measurement

Table 2 shows pre- and post-operative employment status and occupations of the 58 enrolled patients. A significant difference in employment status was seen between before and after surgery (*p* = 0.007).

**Table 2:** Employment status and occupations.

***N =* 58**	**Preop *n* (%)**	**Postop *n* (%)**
**Employment status**		
Level 2 (Working)	42 (72.4)	51 (87.9)
Level 1 (Student or Homemaker)	8 (13.8)	5 (8.6)
Level 0 (No job)	8 (13.8)	2 (3.4)
**Occupations**		
2. Professionals	6 (10.3)	6 (10.3)
3. Technicians and associate professionals	2 (3.4)	3 (5.2)
4. Clerical support workers	2 (3.4)	2 (3.4)
5. Service and sales workers	22 (37.9)	26 (44.8)
6. Skilled agricultural, forestry and fishery workers	1 (1.7)	2 (3.4)
7. Craft and related trades workers	8 (13.8)	12 (20.7)
10. Armed forces	1 (1.7)	0 (0)
Homemaker	5 (8.6)	5 (8.6)
Student	3 (5.2)	0 (0)
Unemployed	8 (13.8)	2 (3.4)

A significant difference between before and after surgery (p = 0.007*). * Significant difference, P < 0.05.

### Secondary Outcome Measurements

#### Neuropsychological Assessment and Employment Status

Table 3 shows the results of neuropsychological assessments before and after surgery in the 28 of the 58 enrolled patients who were able to be evaluated using WAIS-III and WMS-R both preoperatively and ≥3 months after resective surgery. Mean duration from surgery to evaluation was 19.8 months. Significant differences in VIQ, PIQ, and FIQ scores were evaluated by WAIS-III before and after surgery. Among the 28 patients, three patients (one student and two with no job before surgery) obtained employment after surgery. No significant relationships were apparent between post-operative employment status and memory outcomes according to visual (*p* = 0.868) and verbal (*p* = 0.326) memory scales.

**Table 3:** Comparison of pre- and post-operative WAIS- III and WMS-R scores.

***N =* 28**	**Preop**	**Postop**	***P*-value**
**WIAS-III**			
VIQ	87.9 ± 15.3	92.6 ± 17.0	0.014^*^
PIQ	91.8 ± 16.1	96.6 ± 15.1	0.033^*^
FIQ	88.9 ± 15.6	94 ± 16.1	0.005^*^
**WMS-R**			
Verbal memory	89.9 ± 19.2	88.5 ± 21.2	0.69
Visual memory	98.6 ± 13.5	101.2 ± 12.0	0.33

* *Significant difference, P < 0.05*.

*Data are presented as mean ± standard deviation. Abbreviations: FIQ, full-scale intelligence quotient; PIQ, performance intelligence quotient; VIQ, verbal intelligence quotient; WAIS-III, Wechsler Adult Intelligence Scale–III; WMS-R, Wechsler Memory Scale–revised*.

#### Patients With Residual Seizures

Six patients could not obtain freedom from seizures (Table 4). Among these, two patients who were employed before the surgery lost their job and were unable to obtain other employment. One patient who used to be employed at a company changed his job and was hired as a person with disability at a different company. Two patients who were high school students before the surgery were unable to obtain employment and continued living unemployed with their parents. One patient could continue with housework. As a result, only three patients with residual seizures were able to continue with employment.

**Table 4:** Occupations and employment status of patients with residual seizures.

***N =* 6**	**Preop *n* (%)**	**Postop *n* (%)**	
**Occupations**			
5. Service and sales workers	2	1	
9. Elementary occupations	1	1	
Homemaker	1	1	
Student	2	0	
Unemployed	0	3	
**Employment Status**			
Level 2 (Working)	3	2	Job +
Level 1 (Student or Homemaker)	3	1	
Level 0 (No job)	0	3	No job
			*p =* 0.182

## Discussion

In this study, 97% of patients who obtained seizure freedom were able to retain their job. Two of the 58 patients (3%) had no job, a rate similar to the Japanese unemployment rate (3.4% in 2018; https://www.macrotrends.net/countries/JPN/japan/unemployment-rate). From this perspective, being seizure-free appears to represent an important factor behind staying employed. Among the 42 patients (72%) who could continue with employment, 34 patients could stay in the same job, but eight patients (19%) changed their job or employer. We are unable to determine in the present study whether there were negative or positive impacts in these eight patients. However, in addition to the fact that 42 patients (72%) did not lose their jobs, six patients (10%) obtained new jobs, and three students obtained jobs after graduation, suggesting that seizure freedom contributed to increased social participation.

Lifetime employment and a seniority-based wage system have remained fairly well-entrenched in Japan, although recent international trends have slowly been changing the system. In the Japanese system, which involves hiring workers directly out of high school or college and retaining them until mandatory retirement, employer-employee relationships have been likened to the strong bonds of family or parent-child relationships (22, 23). Typical Japanese businesspeople who work for these types of companies used to secure lifetime employment and seniority-based wage structure, but nowadays due to a prolonged economic recession and globalization, the context has been changing. It is not uncommon for many individuals to stay at the same company, as changing jobs is not always advantageous (24–26). The concept of employment continuity is thus important in Japan. This concept could be also important in many East-Asian capitalist countries, as many have used similar systems (25, 27). Therefore, while many studies have assessed relationships between employment and seizure outcomes in Western countries, as shown in Table 5, little information has been accumulated regarding job continuity.

**Table 5:** Other studies assessing the relationship between employment status and seizure outcome in patients after temporal lobe surgery.

**References**	**Follow-up, years range mean (SD) or (range)**	**Patients, *n***	**Age at surgery, years mean (SD) or (range)**	**Employment outcomes**	**Significant factors for employment outcomes**
Sperling et al. (4)	3.5–8.0	35 (47.9%)	SF: Group 1, 31.7 (7.4)	Employment status improved (n): Group 1, 17; Group 2, 7; Group 3, 1	Younger age at surgery seizure outcome
		20 (27.4%)	Some SF: Group 2, 35.4 (7.9)		
		18 (24.7%)	CS Group 3, 33.7 (9.6)		
Reeves et al. (9)	4.2 (2.5–6.5)	134	31	Full- or part-time work gain (8%), loss (11%)	Student or having full-time work before surgery driving after surgery obtaining further education after surgery
Jones et al. (10)	5.8 (2.1)	84 (surgery 63, medical 21)	Surgery 31.3 (8.9) medical 34.7 (10.3)	Full-time work (n) surgery 34 (56%) to 42 (69%) medical 11 (48%) to 9 (39%)	SF state was associated with driving, but not with employment, independent living, or financial independence
Reid et al. (28)	10.3 (1.97)	67	41.4 (9.38)	employed postsurgery SF 27 (79%), CS 12 (36.7%)	Significant differences (SF vs. CS) employment status, driving anxiety, depression, self-esteem, mastery, stigma, affect balance, self-reported health, and quality of life
Paglioli (29)	2–11	134	31.6 (8–62)	Fully employed patients increased 72 (54%) to 102 (76%)	No statistical analysis between SF and employment outcome
Asztely et al. (30)	12.4 (8.6–16.2)	70 (temporal 54)	16.7 (1–58)	Working, n (presurgical, long-term follow-up) SF 31 (82%), 28 (74%) CS 617 (63%), 8 (30%)	Significant differences for employment status between patients with SF and with CS
Benifla et al. (31)	median, 12 (10–20)	42	median, 12.5 (8 months to 18.8 years)	Employment and school enrolment Engel I, II: 24 (86%); Engel III, IV: 8 (57%)	Significant differences for employment and school enrolment between patients with Engel I/II vs. III/IV
George et al. (32)	4.9 (3–8.6)	172	33.67 (18–61)	unemployed before surgery, employed after surgery employed before surgery, unemployed after surgery 23 (18.1%), 14 (11.0%)	Younger age, shorter duration of epilepsy longer post-surgery follow-up (surgery did not significantly improve employment status, but skilled work significantly increased)
Zarroli et al. (13)	9.7 (4–23)	369	35.9 (10–68)	Full- and part-time workers increased 50.7–64.5% of all patients	Working before surgery greater percentage of seizure-free years (younger age, early onset for unemployed and homemakers)
Edelvik et al. (33)	2	473 (temporal 77.8%)	36.5 (10.9)	Full-time employment in seizure-free patients at 2-, 5-, and 10-year follow-up=79%, 79%, 57%	Multivariate predictors: employed preoperatively, favorable seizure outcome, and younger age
	5	220 (temporal 75.5%)	37.7 (10.9)		
	10	240 (temporal 82.1%)	37.1 (11.2)		

*SF, seizure-free; CS, continuous seizures; SD, standard deviation*.

Most studies have reported that good seizure outcomes improved post-surgical employment status. Employment before surgery, younger age at surgery and shorter duration of epilepsy are also significant predictors of good employment status (4, 28, 30, 31, 33). However, George et al. (32) reported that pre- and post-surgical employment status did not differ significantly, although a significant increase was apparent for skilled workers.

The post-operative IQ of patients in this study improved, which might contribute to job retention. However, as we did not analyze correlations between job continuity and improvements in IQ, confirmation of this correlation was beyond the scope of our investigation.

As study limitations, this cross-sectional, observational, non-randomized study was carried out as a retrospective electronic chart review of patients with surgically treated temporal lobe epilepsy in a single institution, and the outcomes were inevitably influenced by the small cohort size, retrospective bias, and regional differences. As differences seem to exist between developed and developing countries (34, 35), further investigation of job continuity after epilepsy surgery warrants an international, multicenter study.

## Conclusion

Seizure freedom could be a factor that facilitates job continuity, but additional data are needed to support that assertion. Further investigation of job continuity after epilepsy surgery warrants an international, multicenter study.

## Data Availability Statement

The original contributions presented in the study are included in the article/supplementary material, further inquiries can be directed to the corresponding author/s.

## Ethics Statement

The studies involving human participants were reviewed and approved by The ethics committee at our hospital approved this study protocol in accordance with the principles of the Declaration of Helsinki (permission number: 3349). The patients/participants provided their written informed consent to participate in this study.

## Author Contributions

TN, AF, KN, SB, HE, KS, TYamaz, and TO: acquisition of data. TN, AF, TO, and HE: analysis and interpretation of data. TN, AF, and TYamam: epilepsy surgery. All authors contributed to the article and approved the submitted version.

## Conflict of Interest

The authors declare that the research was conducted in the absence of any commercial or financial relationships that could be construed as a potential conflict of interest.
